# Tracing the history of a heart

**DOI:** 10.7554/eLife.89988

**Published:** 2023-07-14

**Authors:** Brian Spurlock, Li Qian

**Affiliations:** 1 Department of Pathology and Laboratory Medicine, McAllister Heart Institute, University of North Carolina Chapel Hill United States

**Keywords:** cardiac development, pluripotent stem cells, cardiomyocyte, lineage tracing, genetic engineering, transcription factors, Human, Mouse

## Abstract

Newly developed tools will help researchers understand how the human heart develops and build better models to study and treat congenital heart disease.

**Related research article** Galdos FX, Lee C, Lee S, Paige S, Goodyer W, Xu S, Samad T, Escobar GV, Darsha A, Beck A, Bak RO, Porteus MH, Wu S. 2023. Combined lineage tracing and scRNA-seq reveals unexpected first heart field predominance of human iPSC differentiation. *eLife* **12**:e80075. doi: 10.7554/eLife.80075.

In 2007, one of us lost a cousin after complications from a congenital heart defect. Doctors first realized there was a problem at the 20 week routine heart evaluation. After some initial ambiguity over the diagnosis, it became clear Wayne Preston had Hypoplastic Left Heart Syndrome (HLHS), and the left side of his heart would not be functional (Connor and Thiagarajan, 2007). Although Wayne had lost a second cousin to the same condition some 11 years earlier, the pre- and post-natal surgeries involved in treating HLHS had improved significantly since then, and doctors were cautiously optimistic. But Wayne never left the hospital. He was 19 days old when he died.

Heart defects affect almost 1 in 100 live births worldwide (van der Linde et al., 2011; Koerten et al., 2016), but studying congenital heart disease – and, more broadly, the development of the human heart – is both challenging and ethically fraught (Tan and Lewandowski, 2020). Current regulations mean that in most circumstances, in vitro cultures of human embryos can only be grown for up to two weeks. Furthermore, access to embryonic tissues that are less than six weeks post-fertilization is limited by both regulation and technical hurdles associated with collecting tissue from pregnancies terminated early (Tyser et al., 2021). Therefore, scientists largely rely on non-invasive imaging, ultrasound techniques, and useful but imperfect animal models (Hikspoors et al., 2022; de Soysa et al., 2019). Consequently, very little is known about how the human heart develops between two and five weeks post-fertilization.

The discovery in 2006 that mature differentiated cells can be reprogrammed into an embryonic-like state under certain conditions offered a revolution in the study of development (Takahashi and Yamanaka, 2006). These ‘induced pluripotent stem cells’ (iPSCs) can be programmed to become any type of cell in the body, including heart cells, offering an alternative method to study the development of the human heart in vitro. Now, in eLife, Sean Wu and colleagues – including Francisco Galdos as first author – report a new method for tracking the lineage of iPSCs as they differentiate into muscle cells of the heart (Galdos et al., 2023).

The team (who are based at Stanford University, University of California San Diego School of Medicine and Sungkyunkwan University) differentiated iPSCs into cardiac muscle cells using a common protocol involving the Wnt signaling pathway, which is crucial for normal heart development in vivo (Mandal et al., 2017; Andersen et al., 2018). Cardiac development proceeds in several stages, with the first and second heart fields arising first, shortly after gastrulation (Figure 1A). The first heart field generates the left ventricle (the part of the heart that does not develop correctly in HLHS), while the second heart field generates the right ventricle as well as the outflow tract which helps to transport blood out of the heart (Tan and Lewandowski, 2020; Zhang et al., 2021).

**Figure 1.**
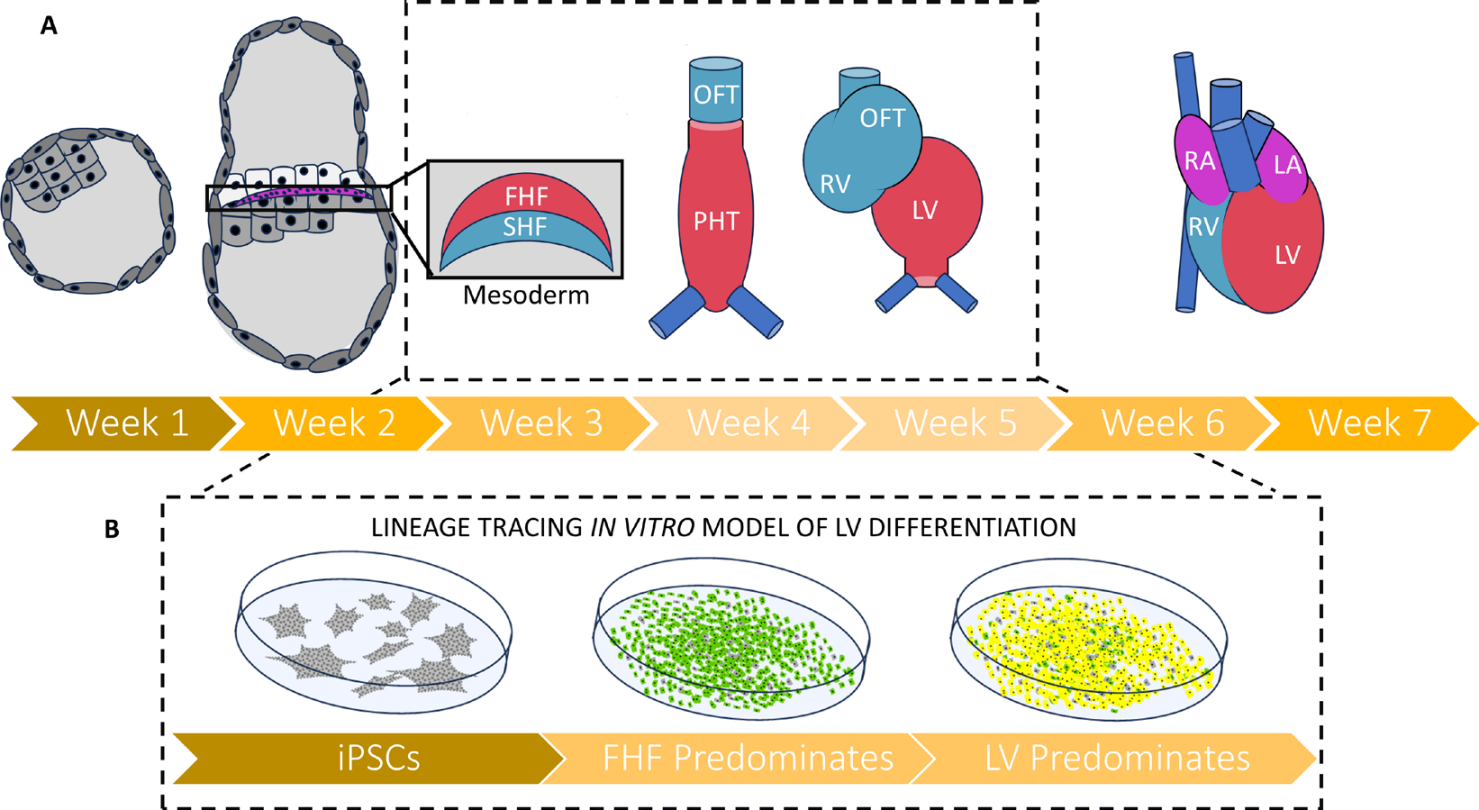
A new strategy for tracing the fate of iPSCs as they develop into muscle cells of the heart. (**A**) Timeline (in weeks post-fertilization) of early human embryonic heart development from the blastocyst through to the emergence of the mesoderm and first (red) and second (blue) heart fields. These each give rise to different parts of the four-chambered heart: the first heart field will go on to form the primitive heart tube and left ventricle, while the second heart field cell will go on to form the right ventricle and the outflow tract. The period of development between two and five weeks post fertilization (depicted in inset) remains understudied in humans due to the 14day rule governing how long human embryos can be grown in the laboratory, as well as the low availability of fetal and embryonic tissues that are less than six weeks post-fertilization. (**B**) Galdos et al. developed a robust lineage tracing strategy to evaluate iPSCs that had been differentiated into cardiac cells by modulating the Wnt pathway. Under this approach, cells transitioning into first heart field cells fluoresce green, and those that terminally differentiate into ventricle muscle fluoresce red. Most cells displayed both fluorescent proteins (resulting in a yellow color), suggesting that Wnt-based differentiation predominately generates muscle cells of the left ventricle. Abbreviations: FHF – first heart field; SHF – second heart field; OFT – outflow tract; PHT – primitive heart tube; RV – right ventricle; LV – left ventricle; RA – right atrium; LA – left atrium; iPSC – induced pluripotent stem cell.

Using CRISPR/Cas9 technology, Galdos et al. developed a fluorescent reporter system which can track iPSCs that have transitioned into cells of the first heart field to eventually become muscle cells of the left ventricle. This was accomplished by adding genes for two reporter proteins. The first, a red fluorescent protein, is paired to a gene expressed in ventricular muscle cells. The second, a green fluorescent protein, is permanently turned on when an established marker of the first heart field is activated, with this activation remaining even after the marker gets deactivated later in the differentiation process (Figure 1B). Surprisingly, 95% of differentiated cells displayed both fluorescent reporters, suggesting that nearly all the iPSCs had developed into left ventricle muscle cells. Even after a more detailed examination, there was no significant trace of cells from the second heart field, suggesting that Wnt-based cardiac differentiation of iPSCs only produces muscle cells of the left ventricle.

Recent methods that can differentiate mouse and human embryonic stem cells into three-dimensional cardiac organoids have led to low but detectable proportions of second heart field cells (Pezhouman et al., 2022; Tampakakis et al., 2019). Growing iPSCs in three-dimensional cultures and organoids, rather than as a two-dimensional layer, may aid the differentiation of the second heart field by allowing cells to form structures akin to those in vivo*,* which could open paths to new cell fates. To test this possibility, Galdos et al. analyzed a dataset showing the genes expressed in the individual cells of cardiac organoids grown from iPSCs (Drakhlis et al., 2021). The analysis showed clusters of cells that appeared to have arisen from the second heart field, including a group that expressed a pattern of genes associated with muscle cells in the outflow tract.

Lineage tracing tools – like the one developed by Galdos et al. – can be used to see how cells respond to specific stimuli over the course of cardiac development, which will be crucial for screening drugs and testing treatments for congenital heart defects. Combining the approach created by Galdos et al. with tools that can trace the fate of other cells in the heart – such as second heart field cells, atrial muscle cells and non-muscle cells – will also allow researchers to rapidly evaluate differentiation protocols used in the laboratory and compare them to in vivo conditions. This could lead to new insights into cardiac development, such as how cells transition from one state to the next, whether some human cells proceed down previously unexplored differentiation pathways and how to direct these pathways to benefit patients. Researchers could then use these findings to generate more efficient strategies for developing heart muscle cells in the laboratory that more accurately reflect the composition of cell types found in the human heart.

Generating iPSCs from patient cells has become invaluable in understanding molecular mechanisms of disease and evaluating potential treatments. Being able to generate iPSCs from patients with HLHS, and track how these cells differentiate into cardiac muscle cells, could reveal what is causing the left ventricle to develop incorrectly. If we, as scientists, are able to achieve this goal, we may be able to prevent future mothers, fathers, sisters and cousins from losing family members to this life-threatening disease.
